# Recognition of 16–18-Year-Old Adolescents for Guiding Physical Activity Interventions: A Cross-Sectional Study

**DOI:** 10.3390/ijerph17145002

**Published:** 2020-07-11

**Authors:** Sunbal N. Bhatti, Emma Watkin, James Butterfill, Jian-Mei Li

**Affiliations:** 1 School of Biological Sciences, University of Reading, Reading RG6 6AS, UK; jian-mei.li@reading.ac.uk; 2 Faculty of Academic Studies, Farnborough College of Technology, Farnborough GU14 6SB, UK; e.watkin@farn-ct.ac.uk; 3 Sports Coaching Department, North Kent College, Gravesend, Kent DA12 2JJ, UK; jamesbutterfill@northkent.ac.uk

**Keywords:** adolescence, health, obesity, physical activity, sedentariness, cardiovascular disease risk

## Abstract

Adolescence is a rapid life stage requiring special attention wherein personal autonomy is developed to govern independent lifestyles. Unhealthy lifestyles are integral to prevailing adolescent physical inactivity patterns. Understudied 16–18-year-olds were investigated to establish physical activity prevalences and influencing health-related lifestyle factors. Adolescents were recruited randomly across 2017–2019 from Farnborough College of Technology and North Kent College, UK. Demographic and health-related lifestyle information were gathered anonymously and analysed using SAS^®^ 9.4 software. Among the 414 adolescents included (48.3% male and 51.7% female), the mean (standard deviation (SD)) age was 16.9 (0.77). Approximately 15.2% smoked and 20.8% were overweight/obese. There were 54.8% perceiving themselves unfit and 33.3% spent >4 h/day on leisure-time screen-based activity. Around 80.4% failed to meet the recommended fruit/vegetable daily intake and 90.1% failed to satisfy UK National Physical Activity Guidelines, particularly females (*p* = 0.0202). Physical activity levels were significantly associated with gender, body mass index, smoking status, leisure sedentary screen-time, fruit/vegetable consumption and fitness perceptions. Those who were female, overweight/obese, non-smoking, having poor fitness perceptions, consuming low fruit/vegetables and engaging in excess screen-based sedentariness were the groups with lowest physical activity levels. Steering physical activity-oriented health interventions toward these at-risk groups in colleges may reduce the UK’s burden of adolescent obesity.

## 1. Introduction

Adolescence is a fast-paced yet crucial life stage which bridges the transition between the dependencies in childhood to the independencies in adulthood wherein behaviours are effortlessly influenced. Cultivation of an unhealthy lifestyle during this period presents a major global health challenge in part owing to the cementing of such behaviours into adulthood [1]. As a consequence, chronic health-deteriorating behaviours manifest as obesity and cardiovascular complications which comprises the leading cause of mortality worldwide (ischaemic heart disease), thereby restricting longevity [2]. Insufficient physical activity, sedentariness and a poor diet are principle mediators of an obesogenic lifestyle [3]. Accordingly, the UK government has defined the National Physical Activity Guidelines (NPAG) for 5–18-year-olds, which advocate at least 60 min of physical activity per day (7 h/week), for an optimal health advantage [4]. However, comprehensive studies report that the NPAGs were largely unmet among those aged 11–15 years old [5] and ~3 million of ≥18-year-olds in the UK [6]. The World Health Organization (WHO) defines adolescence as individuals of the ages 10–19 years. In the realm of physical activity research in the UK, a crucial age (16–18 years) within adolescence is consistently omitted. This research gap has also been acknowledged by the National Centre for Sport and Exercise Medicine in an important report of physical activity prevalences and statistics, used extensively across the nation by policy makers, commissioners and practitioners [7]. Since the development of intellect, skills and changes in lifestyle behaviours are initiated across all ages of adolescence [8], bridging this gap is pivotal to support the targeting of initiatives for health behaviour change.

On account of the adoption of Westernised lifestyles, mounting rates of adolescent obesity have manifested in the current era, exhibiting patterns of incline since 1993 [9], bringing to the surface a crucial public health enigma. Among the regions, the southeast is the most populous in the UK [10], wherein almost a third of those aged ≥16 are obese [11]. Importantly, its prevalence in British youth aged 10–24 years was the highest when compared with 14 European comparator countries [12]. It is widely recognised that failure to achieve NPAG is a principle contributor to the obesity epidemic in the 21st century [13].

Adolescents aged 16–18 experience a diversity of constraints lifted after school age, from parental restrictions to those within the schooling system. As they embark upon college, apprenticeships/traineeships, and/or work, the exposures and influences concurrently change wherein the strict school regulations are lifted. In the development of their personal autonomy, microenvironment influences and their heightened self-consciousness play a pivotal role in pursuing certain health behaviours, for instance, freedom to purchase a range of inexpensive, fat-rich foods suited to personal taste, or effortless attraction to interactive mobile/computer activities without limits (i.e., social media), which encourages excessive sedentary screen-based activity. Moreover, with the increasing expectations of post-school lives, multiple pressures are experienced (including social pressures, work-load and financial) which may reorient their behaviours and advocate injurious and lasting physically inactive lifestyles [1]. Indeed, transitionary life stages are described as obstacles to physical activity [14], which coincide with other adverse health behaviours. As we have previously reported, this holds true in large environments, such as educational institutions [15], as it exposes youth to targeted advertising and various societal influences. For UK adolescents between 16 and 18 years, it is mandatory to engage in some form of education in these settings. Furthermore, students in the southeast of England are prone to adopting unhealthy lifestyles compared to other regions [16]. Collectively, it is plausible that adverse health-related behaviours in adolescents in this region influence their physical activity levels. Since overwhelming evidence exists for the health benefits of physical activity [17], the investigation of health behaviour interaction with physical activity in 16–18-year-olds is of significant value.

Despite the initiative of governmental bodies to invest in this physical inactivity dilemma [18], minimal information describes the (1) physical activity levels and (2) demographic and health-related lifestyle factors that influence levels of physical activity of UK adolescents of 16–18 years. We hypothesise that, coinciding with insufficient physical activity levels, certain demographics and health-related behaviours are influential in the levels of physical activity in 16–18-year-old adolescents in the UK. The aims were to establish physical activity prevalences using NPAG achievement across genders and the health-related lifestyle factors influencing physical activity levels. Herein, we shed novel insight regarding potentially high-risk adolescents, which contributes to the establishment of prevalences in this understudied population. Moreover, the inferences may assist policy makers in steering health interventions to targeted groups to lower cardiovascular disease risk for thousands of UK adolescents.

## 2. Methods 

### 2.1. Study Design

The study was provided with ethical approval by the Faculty Ethics Sub-Committee of University Centre Farnborough, UK, according to their institutional procedures. Study information, including consent forms, were provided to participants prior to the dissemination of surveys. The anonymous multiple-choice design of the questionnaires permitted collection of self-filled data of each participant, including demographic data comprising gender, age, anthropometrics, ethnicity, medical conditions and home country of residence. Further, health-related lifestyle behaviours were collected which comprised: (1) quantity and frequency of cigarette smoking (cigarettes/week) categorised into smokers and non-smokers and, of those who were ≥18 years old, quantity and frequency alcohol consumption (units/week) which was calculated using the formula: strength (alcohol by volume measured as a percentage of alcohol) x volume (ml) ÷ 1000 = units of alcohol; (2) the duration spent in physical activity (h/week) which were ordered in to the categories: 0.25–2.5, 2.5–8 or >8 h/week in compliance with NPAG [4]; (3) meal frequency (meals/day) and fruit/vegetable portions consumed (portions/day); (4) sleep duration (h/day) which was separated into <7 and ≥7 h/day in agreement with the recommended hours for young adults [19]; (5) leisure-time sedentariness on screen-based activities including computer use, watching television (TV) and gaming (≤4 and >4 h/day) and; (6) fitness self-perceptions (fit or unfit) and barriers to physical activity (open-ended). The body mass index (BMI) was categorised in keeping with the guidelines defined by the National Institutes of Health [20] as follows: underweight (BMI <18.5 kg/m^2^); normal weight, (BMI 18.5–24.9 kg/m^2^) and overweight/obese (BMI ≥25/≥30 kg/m^2^).

### 2.2. Participant Recruitment and Data Collection

Recruitment of volunteers took place during the years of 2018 and 2019 at both North Kent College, covering regions of Dartford, Gravesend and Kent (UK), and Farnborough College of Technology spanning regions of Surrey, Aldershot and Farnborough (UK). Recruitment was achieved by multi-stage probabilistic sampling using the 4000-student college census as a sampling frame and sampling randomly within departmental strata and then randomly sampling within classes, as our study intended to obtain a representative sample of UK college students for the southeast region of England. The inclusion criteria were students aged 16–18 years, within two years of college life and without documented medical conditions. Exclusion criteria were having a disability or documented medical condition which would otherwise limit the engagement in physical activity (i.e., bone/joint disorders), being above the age of 18 and being incomplete survey entries. Data were entered electronically for statistical analysis. This study was given favourable ethical opinion by the Faculty Ethics Sub-Committee of University Centre Farnborough (11–05-18), UK.

### 2.3. Statistical Analysis

Statistical analysis was performed using SAS software, Version 9.4 (SAS Institute Inc., Cary, NC, USA). Anthropometric and lifestyle data are reported as mean (standard deviation (SD)). A multinomial logistic regression was performed examining the associations between the response variable physical activity (0, 0.25–2.5, 2.5–8 and >8 h/week) with gender, ethnicity, BMI, smoking status, alcohol consumption, eating frequency, fruit/vegetable intake, sleep duration, leisure screen-time (computer/TV) and perception of fitness. *p*-values and adjusted odds ratios (OR) were examined to explore parameter influence on physical activity levels. Statistical significance was accepted at 5% using a Wald chi-square test [21]. The use of a binomial logistic regression with dichotomised physical activity levels according to age-specific NPAG [4] was also performed. The results were presented with adjusted OR followed by their 95% confidence intervals for each explanatory variable, in brackets.

## 3. Results

### 3.1. Participant Demographics and Health-Related Lifestyle Behaviours

Among the 414 participants, 200 (48.3%) were males and 214 (51.7%) were females with a mean (SD) age of 16.9 (0.77). Table 1 provides participant demographic information and health-related lifestyle behaviours. The majority (*n* = 401, 96.9%) of participants were European residents and only 13 (3.1%) were from other regions. There were 302 students (72.9%) of a Caucasian ethnicity, 47 (11.4%) were Asian, 17 (4.1%) were Black African and the rest (48, 11.6%) were from other ethnic origins. There were 63 (15.2%) participants who reported to be smokers with a mean (SD) of 5.1 (19.22) cigarettes consumed/week.

The average BMI was 22.6 (4.18) kg/m^2^, a value residing within the normal BMI range (Table 1). Among participants, 86 (20.8%) were overweight/obese, 47 (11.4%) were underweight, and 281 (67.9%) were of a normal weight. More males were overweight (48, 24.0%) than females (38, 17.8%). Of the 108 participants who were 18 years old, 71 (65.7%) reported alcohol consumption with an average (SD) intake of 5.9 (11.85) units/week for males and 5.4 (9.24) units/week for females, remaining below alcohol recommendations of 14 units/week [22]. A greater percentage of 18-year-old females (74.1%) consumed alcohol relative to males (57.4%). 

### 3.2. Physical Activity Levels and Influencing Factors

**Figure 1:**
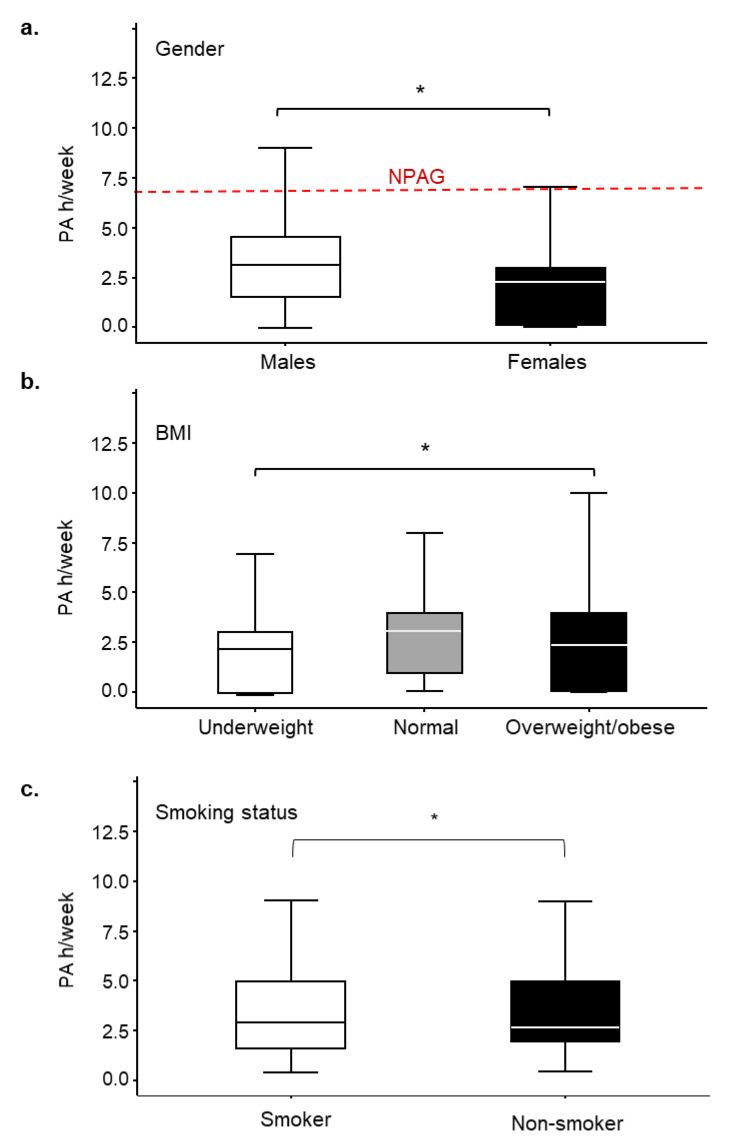
The relationship between the levels of physical activity and gender, body mass index (BMI) or smoking status in 16–18-year-old adolescents (*n* = 414). (**a**) Differences of physical activity levels between males [mean (standard deviation (SD)): 3.23 (2.70), *n* = 200] and females [mean (SD): 2.27 (2.62), *n* = 214]; (**b**) Differences of physical activity levels between BMI categories [mean (SD): underweight 2.18 (2.60), normal 3.00 (2.95), overweight/obese 2.30 (2.42)]; and (**c**) Differences of physical activity levels between smokers [mean (SD): 2.8 (2.83)] and non-smokers [mean (SD): 2.6 (2.55)]. Red dashed line indicates the UK National Physical Activity Guidelines (NPAG) recommended for those between the ages of 5 to 18 years old. Gender * *p*-value 0.0216, odds ratio (OR) 0.61 (0.404, 0.930); BMI (normal and overweight/obese) * *p*-value 0.0038, OR 2.15 (0.951, 4.854); smoking * *p*-value 0.0413, OR 1.82 (1.025, 3.247).

On average, time spent in physical activity was 3.2 (2.70) h/week for males and 2.3 (2.62) h/week for females, substantially lower than the NPAG (7 h/week) [4]. There were 373 (90.1%) participants who failed to meet their age-specific NPAG (Table 1 and Figure 1a). Further, among the total participants, 121 (29.2%) reported failure to engage in any form of physical activity (0 h/week), in which a greater percentage were females (73, 34.1%) in comparison to males (48, 24.0%). Statistical analysis revealed that there were significantly more females (200/214, 93.5%) than males (173/200, 86.5%) who were not satisfying NPAG, *p*-value 0.0202, OR 0.45 (0.228, 0.883) (Table 1). 

Another factor associated with physical activity levels was BMI, *p*-value 0.0038, OR 2.15 (0.951, 4.854). A BMI of ≥25 kg/m^2^ was associated with significantly lower levels of physical activity (Figure 1b). An individual’s smoking status also arose as an influencing factor for physical activity levels, *p*-value 0.0413, OR 1.82 (1.025, 3.247), such that smokers engaged in greater levels of physical activity than non-smokers (Figure 1c).

**Figure 2:**
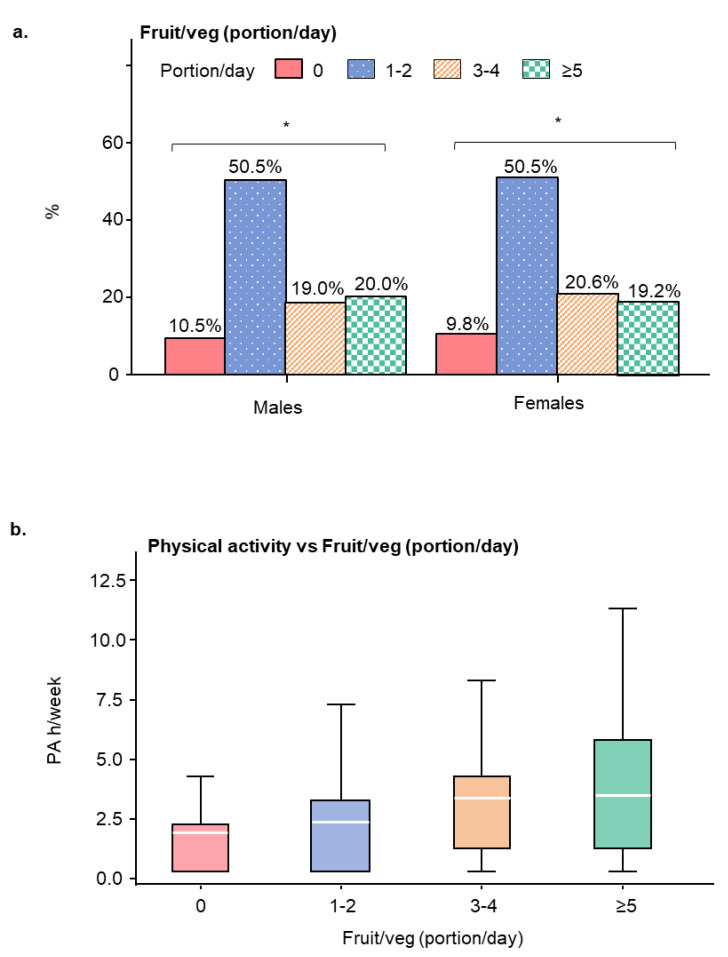
Daily average consumption of fruits and vegetables (portion/day) in participants and its relationship with the levels of physical activity. (**a**) Bar figure displaying the percentages of fruits and vegetables consumed daily by male and female students. (**b**) Relationship between the levels of physical activity and daily fruit and vegetables intake (portion/day). * *p*-value 0.0050, odds ratio OR 3.96 (1.514, 10.374). Data are represented as mean (standard deviation (SD)).

We found that 42 (10.1%) students failed to consume any portion of fruit/vegetables per day. The majority of students (209, 50.5%) consumed 1–2 portions of fruit/vegetables per day, 82 (19.8%) consumed 3–4 portions/day, and 81 (19.6%) consumed ≥5 portions/day with negligible gender differences (Figure 2a). Fruit/vegetable consumption was positively associated with physical activity (Figure 2b), *p*-value 0.0050, OR 3.96 (1.514, 10.374). The mean (SD) value of physical activity for those consuming 1–2 portions/day was 2.3 (2.66) h/week, for those consuming 3–4 portions/day was 3.2 (2.60) h/week and for those consuming more than 5 portions/day was 3.3 (2.84) h/week. The lowest level of physical activity, 1.9 (2.39) h/week, was observed for those who did not have daily intake of fruits/vegetables. Further, we found that the majority of the participants (241, 58.2%) consumed ≥3 meals/day, wherein the number for males (136, 68.0%) was greater than that for females (105, 49.1%) (Table 1). No association was observed between eating frequency and physical activity.

**Figure 3:**
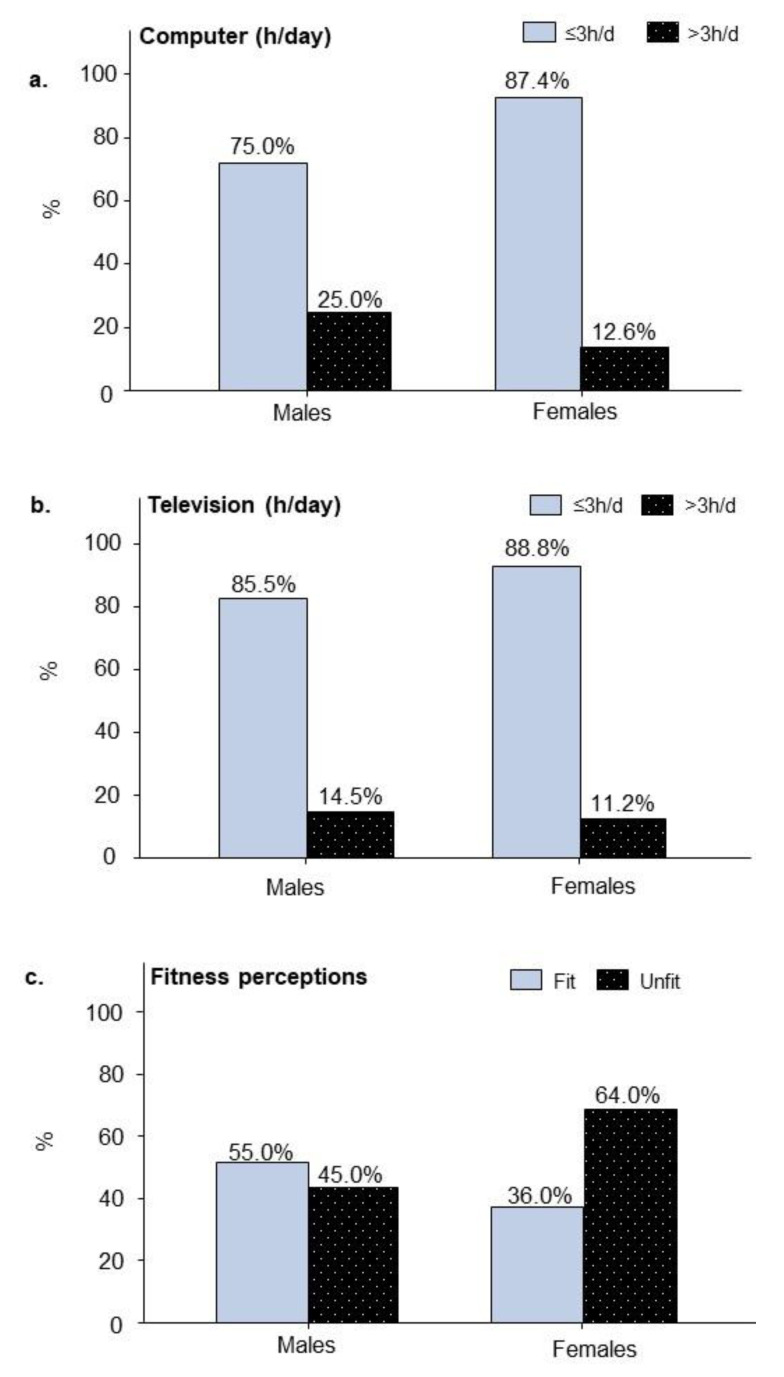
Differences in leisure-time sedentary screen behaviours and fitness perceptions between male (*n* = 200) and female adolescent students (*n* = 214). (**a**) Percentages of students engaged in leisure-time computer activity (h/day). (**b**) Percentages of students engaged in leisure-time television (TV) activity (h/day). (**c**) Percentages of students separated by self-assessed fitness perception. The influence of physical activity levels are given by the *p*-values and odds ratios (OR) that follow: sedentary behaviours including (**a**) computer (h/day), *p*-value 0.0067, OR 1.887 (1.192, 2.985) and (**b**) watching television, *p*-value 0.0052, OR 0.402 (0.213, 0.762); (**c**) fitness perceptions, *p*-value < 0.0001, OR 0.325 (0.208, 0.507).

There were 138 (33.3%) students who spent >4 h/day leisure-time sedentary in computing, gaming or watching movies with more males (78, 39.0%) than females (60, 28.0%). A small percentage of participants spent >3 h/day on the computer (78, 18.8%) or watching television (53, 12.8%) which was a similar proportion across genders, except that nearly twice the number of males (50, 25.0%) spent >3 h/day on the computer than females (27, 12.6%) (Figure 3a,b). Leisure-time screen-based activity was associated with reduced levels of physical activity, *p*-value 0.0065, OR 0.402 (0.213, 0.762).

We also found that 226 (54.6%) participants slept less than the recommendation (<7 h/day) [19]. It appeared that females (120, 56.1%) spent less time in sleep than males (106, 53.0%), though there was no correlation with physical activity.

### 3.3. Perception of Fitness and Barriers to Physical Activity

More than half of all participants (227, 54.8%) perceived themselves as being physically unfit, with more females (137, 64.0%) reporting this in comparison to males (90, 45.0%), (Figure 3c). Fitness perceptions were associated with physical activity, such that those who perceived themselves as unfit had a three-fold reduction in physical activity levels, *p*-value < 0.0001, OR 0.325 (0.208, 0.507) (Figure 3c). There were 175 (42.3%) participants who thought themselves unfit and also failed to meet NPAG, wherein 65 (32.5%) were males and 110 (51.4%) were females. Moreover, there were 89 (21.5%) participants who perceived themselves to be fit yet failed to meet NPAG. The explanations given for not meeting NPAG were “No time” and “Don’t want to” (91.1%), followed by “No facility” (8.5%) and “No money” (0.4%). 

## 4. Discussion

Adolescence is a rapid stage of life requiring special attention [8], wherein personal autonomy is developed for the governance of independent lifestyles. An estimated 738 thousand 16–18-year-old adolescents are required to be trained in UK colleges that provide work, apprenticeships and/or education in an influential environment instilling health-related behaviour modification [23]. Among these changes, insufficient physical activity damages health, persists into adulthood [1] and predicts cardiovascular disease risk [24]. Adopting NPAG improves diverse aspects of health such as cognitive function, disease and longevity [1,2,17]. Health research pertaining to adolescents is heavily focused on school or university ages [25,26,27,28]. Minimal studies explore 16–18-year-olds, which is a key transitionary life stage important in reinforcing lifestyle trajectories, thereby deeming valuable the recognition of their physical activity levels and the behaviours influencing physical activity engagement.

Approaching 50% of students within the southeast of England, a population-dense region, gain weight upon commencing higher education [10,16]. This population is gaining extensive interest by public health policy-makers worldwide for health-intervention, thereby making it particularly constructive to recognise those at risk [12]. A recent study has shown that university students who performed <3 h/week physical activity (any form of exercise or sport) had significantly lower cardiorespiratory fitness (VO_2_max) and heart rate recovery, which put them at risk for cardiometabolic diseases [29]. Herein, we provide novel indication that the prevalence of college students failing to achieve NPAG (90.1%) is of a greater degree than estimated, and to a larger degree for females. According to other reports, recently the lack of physical activity in ~15-year-old adolescents reached 48% to around 65% in various regions of the world [30,31] and rose to around 80% in UK adolescents [5,32]. As we found that 60.8% of students were active yet failed to meet NPAG, we suggest that the intention for physical activity is prevalent, though they fail to reap the health benefit due to insufficient motivation and time. Commonly, females justify physical inactivity due to social pressures, forced competition, time constraints and body dissatisfaction [33]. Agreeing with the latter, we show that more females have poorer self-perceptions of fitness than males which correlated well with their inability to achieve NPAG. Collectively, physical activity educational interventions in college students may be beneficial, particularly for females, and may improve fitness self-perceptions.

Overweight/obesity is an established risk factor for cardiovascular diseases [3]. Herein, a large percentage of the cohort (20.8%) were overweight/obese, particularly males, indicating that the prevalence of obesity may be worse in college students than general figures suggested for UK adolescents (8.1%) [12]. Moreover, mirroring our finding, it is unanimously agreed that BMI is negatively correlated with physical activity in youth [34]. Previously, the consensus was that smoking in adolescence negatively correlated with physical activity [35], since then research has demonstrated that certain forms of physical activity (walking/cycling), when controlled for risk factors (peer influence/substance misuse), enhance the odds of smoking by four-fold [36]. Harmonising with this interaction, herein smokers were more likely to engage in greater levels physical activity than non-smokers. Explanations of this paradoxical interaction bring to surface that underlying motives (weight loss) and/or seeking membership thresholds promotes increased cigarette use [36]. Taken together, educational intervention of specific physical activities which exhibit a negative association with smoking (running/racquet sports) in college students may promote health.

Mounting sedentariness among youngsters poses a risk for the development of cardiometabolic diseases, particularly in obese adolescents [37]. There were 33.3% of participants in this cohort who reported excessive sedentariness which is negatively associated with physical activity levels. Promoting compensatory physical activity between lecture hours and encouraging leisure-time outdoor activity, at minimum engaging in longer durations of low-level activity, could make significant contributions to cardiovascular, neuromuscular, bone and mental health for adolescents [17,38,39].

The WHO recommends ≥5 portions of fruit/vegetables per day as an important dietary component for chronic disease prevention [40], yet we found that the majority (80.4%) of students failed to meet this standard. In agreement with our findings, reports suggest that engagement in physical activity motivates one to practice healthy eating [41]. Conversely, others suggest, through unconscious energy homeostasis, adopting a healthy behaviour in one area may endorse unhealthy behaviours in another area [42]. In view of the prevalence of low fruit/vegetable consumers in our cohort, providing college interventions to advocate the nutritional value of consuming fruit/vegetables and raising health-consciousness may positively influence their physical activity levels. Furthermore, the distribution of an individual’s calorie intake throughout the day influences weight status and researchers suggest a protective effect of increased meal frequency on obesity in the young [43]. Our results, however, reveal more than half (58.2%) of adolescent students consume ≥3 meals/day, though no association existed between meal frequency and physical activity levels. 

There were limitations of the study methodology as anthropometric and lifestyle behaviours were not objectively measured but obtained solely through self-report by participants, which may give rise to inaccuracies. For example, some adolescents may misestimate their weight or physical activity levels, which may affect the associations found herein. Nevertheless, the reliability of survey data was previously demonstrated as valid and reliable [44]. Another limitation is that the participants of this study were recruited from two colleges which may not be representative of the entire population of this age in the UK; however, the geographic distribution of individuals in this study managed to cover several populated suburb regions of southeast England. The ethnicity percentage in this sample closely reflects the characteristics of the general 16–18 student population reported by the Department for Education [45]. Furthermore, the cross-sectional nature of the study prohibits the inference of causality. Although they are prevalent behaviours, it is important to note that the engagement in screen-based activities (computer/TV) may not wholly represent time spent sedentary. However, given the limited information available for the prevalence of physical activity levels of 16–18-year olds in the UK, our study delivers statistics for this research gap and indicates important groups to target for health promotion.

## 5. Conclusions

In closing, the vast majority (90.1%) of 16–18-year-old adolescents in the southeast of England fail to meet NPAG, and 80.4% fail to meet the recommended fruit/vegetable daily intake. We indicate that physical activity is diminished in adolescents who are females, overweight/obese, non-smokers, have poor fitness perceptions, low fruit/vegetable consumption and engage in high screen-based sedentariness. Steering certain forms of physical activity-oriented health interventions toward these at-risk groups in colleges may reduce the UK’s prevailing enigma of adolescent obesity and future cardiovascular outcomes.

## Figures and Tables

**Table 1:** Demographics and health-related lifestyle behaviours by gender.

Variable	Males (*n* = 200)	Females (*n* = 214)	Total (*n* = 414)	*p*-Value ^a^
Age (years) mean (SD)	16.9 (0.78)	16.9 (0.76)	16.9 (0.77)	
Residential status	*n (*%)	*n (*%)	*n (*%)	
Europe	189 (94.5)	212 (99.1)	401 (96.9)	
Outside Europe	11 (5.5)	2 (0.9)	13 (3.1)	0.9844
Ethnicity				
Caucasian	140 (70.0)	162 (75.7)	302 (72.9)	
Asian	29 (14.5)	18 (8.4)	47 (11.4)	
Black African	8 (4.0)	9 (4.2)	17 (4.1)	
Other Ethnic Group	23 (11.5)	25 (11.7)	48 (11.6)	0.2445
BMI (kg/m^2^) mean (SD)	23.2 (4.34)	22.0 (3.96)	22.6 (4.18)	
Underweight (<18.5)	22 (11.0)	25 (11.7)	47 (11.4)	
Normal (18.5–24.9)	130 (65.0)	151 (70.6)	281 (67.9)	
Overweight/Obese (≥ 25/≥ 30 kg)	48 (24.0)	38 (17.8)	86 (20.8)	0.0038
Smoking (cigarette/week) mean (SD)	5.2 (18.31)	5.0 (20.08)	5.1 (19.22)	
Non-smoker (%)	85.0	84.6	84.8	
Smoker (%)	15.0	15.4	15.2	0.0413
Alcohol (units/week) ^b^ mean (SD)	5.9 (11.85)	5.4 (9.24)	5.6 (10.58)	
No alcohol (%)	42.6	25.9	34.3	
Alcohol (%)	57.4	74.1	65.7	0.8410
Eating frequency (meals/day)				
<3	64 (32.0)	109 (50.9)	173 (41.8)	
≥3	136 (68.0)	105 (49.1)	241 (58.2)	0.4093
Sleeping (h/day)				
<7	106 (53.0)	120 (56.1)	226 (54.6)	
≥7	94 (47.0)	94 (43.9)	188 (45.4)	0.8397
Leisure screen-time (h/day)				
≤4	122 (61.0)	154 (72.0)	276 (66.7)	
>4	78 (39.0)	60 (28.0)	138 (33.3)	0.0065
Meeting NPAG (h/week) ^c^				
Not meeting guidelines (<7)	173 (86.5)	200 (93.5)	373 (90.1)	
Meeting guidelines (≥7)	27 (13.5)	14 (6.5)	41 (9.9)	0.0202

^a^*p*-value analysing the influence of physical activity levels with health-related lifestyle factors yielded from the generalised logistic regression. ^b^ Of those who were 18 years old. BMI, body mass index; NPAG, National Physical Activity Guidelines; SD, standard deviation. ^c^ *p*-value as output from the binomial logistic regression analysis.

## Data Availability

The datasets used and analysed during the current study are available from the corresponding author on reasonable request.
